# Human genetics of tuberculosis: a long and winding road

**DOI:** 10.1098/rstb.2013.0428

**Published:** 2014-06-19

**Authors:** Laurent Abel, Jamila El-Baghdadi, Ahmed Aziz Bousfiha, Jean-Laurent Casanova, Erwin Schurr

**Affiliations:** 1 Laboratory of Human Genetics of Infectious Diseases, Necker Branch, Institut National de la Santé et de la Recherche Médicale U1163, 75015 Paris, France; 2 Paris Descartes University, Sorbonne Paris Cité, Imagine Institute, 75015 Paris, France; 3 St Giles Laboratory of Human Genetics of Infectious Diseases, Rockefeller Branch, The Rockefeller University, New York, NY 10065, USA; 4 Genetics Unit, Military Hospital Mohamed V, Hay Riad, 10100 Rabat, Morocco; 5 Clinical Immunology Unit, Department of Pediatric Infectious Diseases, Hospital-University Center Ibn Rochd, King Hassan II University, Casablanca, Morocco; 6 Howard Hughes Medical Institute, The Rockefeller University, New York, NY 10065, USA; 7 McGill International TB Centre, The Research Institute of the McGill University Health Centre, Montreal, Quebec, Canada H3G 1A4

**Keywords:** primary tuberculosis, pulmonary tuberculosis, latent tuberculosis infection, Mendelian predisposition, complex genetic predisposition, genetic variant

## Abstract

Only a small fraction of individuals exposed to *Mycobacterium tuberculosis* develop clinical tuberculosis (TB). Over the past century, epidemiological studies have shown that human genetic factors contribute significantly to this interindividual variability, and molecular progress has been made over the past decade for at least two of the three key TB-related phenotypes: (i) a major locus controlling resistance to infection with *M. tuberculosis* has been identified, and (ii) proof of principle that severe TB of childhood can result from single-gene inborn errors of interferon-γ immunity has been provided; genetic association studies with pulmonary TB in adulthood have met with more limited success. Future genetic studies of these three phenotypes could consider subgroups of subjects defined on the basis of individual (e.g. age at TB onset) or environmental (e.g. pathogen strain) factors. Progress may also be facilitated by further methodological advances in human genetics. Identification of the human genetic variants controlling the various stages and forms of TB is critical for understanding TB pathogenesis. These findings should have major implications for TB control, in the definition of improved prevention strategies, the optimization of vaccines and clinical trials and the development of novel treatments aiming to restore deficient immune responses.

## 1. Introduction

**Figure 1.**
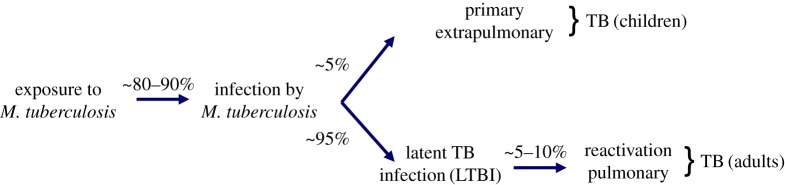
A schematic of the natural history of human infection by *M. tuberculosis,* and the subsequent development of clinical TB. Despite sustained high-level exposure, a substantial proportion of subjects (approx. 10–20%) do not become infected, and hence never develop disease. About 5% of infected individuals develop clinical TB within 2 years of infection; this ‘primary’ TB is particularly common in children, and could be associated with extrapulmonary disease. The remaining persons infected with *M. tuberculosis* develop latent TB infection (LTBI). Only a minority of subjects with LTBI (approx. 5–10%) develop clinical TB during their lifetime, typically owing to reactivation of the original infection. (Online version in colour.)

Tuberculosis (TB) remains a major public health problem, as *Mycobacterium tuberculosis* infects an estimated one-third of the world's population, resulting in approximately 8.6 million new cases of TB and approximately 1.3 million deaths in 2012 [1]. TB bacilli are transmitted by the inhalation of aerosolized droplets generated by the coughing of a patient with active TB. A substantial proportion of subjects do not become infected despite sustained high levels of exposure, as shown by negative tuberculin skin test (TST) and/or interferon (IFN)-γ release assays (IGRAs), and hence never develop disease (figure 1). About 5% of infected individuals develop clinical TB within 2 years of infection, either without latency or after a very short latent phase (figure 1) [2,3]. This ‘primary’ TB is particularly common in children, running an acute course and often associated with extrapulmonary disease owing to dissemination of the bacillus in the bloodstream [4,5]. However, most people infected with *M. tuberculosis* develop latent TB infection (LTBI). LTBI is characterized by positive TST and/or IGRA and an absence of overt clinical signs (figure 1) [2,6,7]. Most subjects with LTBI (approx. 90–95%) never develop clinical disease. The remaining 5–10% develop clinical TB later in life, typically owing to reactivation of the original infection. This ‘reactivation’ TB is predominantly a chronic pulmonary disease of adults, resulting in extensive lung damage and the efficient airborne transmission of bacteria. Hence, individuals susceptible to infection display three clinical presentations: (i) not entering LTBI (primary TB), (ii) remaining with LTBI (silent infection) or (iii) exiting from LTBI (reactivation TB).

Clinical and epidemiological surveys conducted since the 1910s have provided strong evidence that each step underlying infection or disease is controlled by host genetic factors [8–10]. Familial aggregation studies have provided the most convincing evidence [9,10]. In families with an index sputum-positive TB patient, spouses with family histories of TB were found to develop manifest TB more frequently than those without such histories [10]. Twin studies have also shown much higher concordance rates for monozygotic than for dizygotic pairs for clinical TB (combining primary and reactivation TB) [10,11]. In the seminal study conducted by Kallmann and Reisner in New York State, 308 twin pairs in which one of the pair was a confirmed index TB case were studied [11]. In this sample, the percentage of twin siblings of index cases developing manifest TB (defined on the basis of clinical, chest X-ray and sputum examinations) was 66.7% for monozygotic twins (52/78) and 23% (53/230) for dizygotic twins [11]. Interestingly, similar percentages were obtained when the sample was subdivided into groups on the basis of the known history of exposure of the twin siblings to a known active TB patient (table 1). It has long been known that the incidence of TB is particularly high in newly exposed populations [9], such as African populations and native Americans [12]. Similarly, the susceptibility to *M. tuberculosis* infection of an exposed individual, as measured by the TST, has been shown to be correlated with the region of ancestry of the individual concerned [13,14]. As for clinical TB disease, familial studies, including twin studies, have shown TB infection phenotypes (mostly TST result, considered as a quantitative trait) to be highly heritable (more than 50%, as detailed in §2).

**Table 1.** Proportion of monozygotic and dizygotic twins of index cases with TB, as a function of the history of exposure of the co-twin to a known active TB patient, from the study by Kallmann & Reisner [11].

	monozygotic co-twins			dizygotic co-twins		
	number of TB cases	total number	% of TB cases	number of TB cases	total number	% of TB cases
history of exposure	36	52	69.2	46	175	26.3
without known exposure	16	26	61.5	7	55	12.7
total	52	78	66.7	53	230	23.0

Furthermore, a long series of experimental studies in various animal models, beginning in the 1930s, has also established the importance of host genetic background for determining the outcome of infection with *M. tuberculosis* (reviewed in [7,15,16]). In mice, a key antimycobacterial locus (the *Bcg* locus) has been shown to be allelic to *Nramp1* [17]. Knockout mice showed that CD4 T cells were required for immunity to *M. tuberculosis* and that the three most important antimycobacterial molecules required for *M. tuberculosis* destruction by phagocytes were tumour necrosis factor (TNFI-α), IFN-γ and nitric oxide synthase 2 [7,15,18,19]. Human studies have confirmed most of these findings through demonstrations of an increase in the risk of TB in AIDS patients [20,21], in patients on anti-TNF treatment [22], and in patients with genetic defects impairing IFN-γ immunity [8,23,24]. These studies have clearly demonstrated the critical importance of CD4-mediated immunity and of the interleukin (IL)-12/IFN-γ loop in baseline resistance to *M. tuberculosis*, but the reasons for which individuals otherwise displaying full immune competence develop TB remain largely unknown. In this context, the identification of genetic variants increasing the risk of TB constitutes a powerful approach to deciphering the mechanistic basis of TB pathogenesis. We provide here an overview of the principal human genes known to underlie the interindividual variability of susceptibility at each of the three main steps in the natural course of *M. tuberculosis* infection. We also discuss the contribution of genetic research to the development of new approaches to combat TB.

## 2. Genetic control of tuberculosis infection

There is no direct test for infection with *M. tuberculosis* and the phenotype of *M. tuberculosis* infection is inferred exclusively from quantitative measurements of antimycobacterial immunity. These assays cannot distinguish a possible anamnestic response to *M. tuberculosis* from persistent infection with the bacillus. The TST is the most widely used method [25]. The skin induration generated by the TST is caused by the accumulation of histiocytes and T cells around intradermal deposits of *M. tuberculosis* antigens. More recently, two *in vitro* blood assays, measuring either the secretion of IFN-γ by lymphocytes or the frequency of IFN-γ producing blood cells in response to *M. tuberculosis* antigens (IGRAs), have been developed [26]. TST and IGRAs assess different aspects of antimycobacterial immunity and are not fully concordant in predicting infection with *M. tuberculosis* [27]. Little or no reactivity in these tests in individuals exposed to *M. tuberculosis* is indicative of innate resistance to *M. tuberculosis* infection. In household studies, 30–50% of contacts with heavy short-term exposure do not become infected [28,29], revealing substantial heterogeneity in susceptibility to infection. We and others have focused on TST and IGRA results as quantitative traits and have shown heritability to be high for the results of both tests following exposure to *M. tuberculosis*. In Gambia, a study of healthy twins estimated the heritability of TST responsiveness and quantitative IGRA reactivity at 71% and 39%, respectively [30]. The heritability of quantitative TST reactivity in young healthy children exposed to an active TB case has been estimated at 92% in Chile [31]. In a South African familial sample from an area of hyperendemic TB disease, the heritability of quantitative IFN-γ release responses was estimated to be between 43 and 58%, depending on the nature of the stimulating antigen [32]. Likewise, the heritability of the frequency of antigen-specific IFN-γ^+^CD4^+^ and IFN-γ^+^CD8^+^ has been estimated at 53–74% [32]. A complex segregation analysis of TST reactivity in related household contacts of TB index cases in a Colombian population provided evidence for a major codominant gene accounting for approximately 65% of TST variability [33]. By contrast, a family study conducted in Uganda reported a lower estimate of heritability for IGRA responses (approx. 17%) [34]. However, as IGRA reactivity was adjusted for TST response in this study, this lower estimate of heritability may reflect genetic components common to the TST and IGRA responses. More recent data from Uganda carefully adjusted for shared environment yielded higher estimates for the heritability of IGRA response (approx. 30%) [35].

Despite the strong evidence in favour of an impact of genetic factors on the assays used for LTBI, only a small number of studies have aimed to identify the genetic variants underlying susceptibility to *M. tuberculosis* infection. TST reactivity was the phenotype studied in all these studies. Candidate gene association studies have focused on TST response as a binary trait, defined according to various thresholds (0, 5 or 10 mm). In a large study of 3622 TST-positive individuals and 244 TST-negative healthy controls in Ghana, an *IL10* promoter haplotype (−2849A/−1082A/−819C) was found to be significantly more frequent in TST-positive than in TST-negative subjects (15.3% versus 9.7%, odds ratio (OR) = 2.09 (1.2–3.5), *p* = 0.012) [36]. This haplotype was also associated with low levels of circulating IL-10, suggesting a role for IL-10 in the initial host response to *M. tuberculosis*. However, the similarity of age distribution between TST-negative and TST-positive subjects was not shown. Consistent with the findings for the sample from Ghana, the prevalence of TST negativity was found to be 1.5 times higher in individuals carrying the high-level IL-10-producing genotype GG at single nucleotide polymorphism (SNP) −1082A>G than in individuals carrying the AA and AG genotypes, in an indigenous population from Brazil [37]. Additional associations of cytokine genes with TST reactivity in the Brazilian sample have yet to be replicated.

In Uganda, a genome-wide (GW) linkage analysis reported results suggesting that persistent TST negativity (defined as a TST < 10 or 5 mm, according to age and HIV status) was linked to chromosomal regions 2q21–2q24 and 5p13–5q22 [38]. A study of TST reactivity in a sample of multiplex families from South Africa identified two major loci affecting TST-positivity *per se* (*TST1*) and the intensity of TST reactivity (*TST2*) [39]. *TST1* was identified by focusing on the phenotype of TST positivity versus TST negativity (i.e. TST = 0), and maps to chromosome 11p14 (lod score = 3.81, *p* = 1.4 × 10^–5^). A second phenotype studied was the size in millimetres of the skin induration in TST-tested subjects. The size of the induration, considered as a quantitative trait, was impacted by a locus on chromosome 5p15 which was termed *TST2* (lod score = 4.00, *p* = 9 × 10^–6^). The most parsimonious explanation for the role of these two loci is that *TST1* reflects innate resistance to infection with *M. tuberculosis* whereas *TST2* reflects T-cell-mediated antimycobacterial immune responses. Unexpectedly, it was subsequently discovered that a locus affecting the production of TNF by blood cells in response to bacillus Calmette–Guérin (BCG) and BCG plus IFN-γ, *TNF1*, is genetically indistinguishable from *TST1* [40]*.* This raises the exciting possibility that innate resistance to *M. tuberculosis* infection may involve a TNF-mediated effector mechanism. Such a possibility dovetails neatly with the function of TNF in macrophage activation during early stages of infection. No GW association study (GWAS) has yet been performed for *M. tuberculosis* infection.

## 3. Genetic control of severe primary tuberculosis

About 5% of infected individuals develop clinical TB within 2 years of infection (figure 1), either without latency or after a very short latent phase [3]. This ‘primary’ TB is particularly common in children, some of whom develop a haematogenous disseminated form (referred to here as ‘severe primary TB’) [2]. Severe primary TB was, by far, the most frequent form in children in areas of endemic disease before BCG vaccines and antimycobacterial antibiotics became available, resulting in high rates of mortality in children under the age of 2 years [9,23,41]. The risk of severe primary TB remains highly dependent on age at primary infection, decreasing from 10 to 20% for children under the age of 1 year to less than 0.5% for children over the age of 5 years [4,5]. These severe forms are mostly either miliary or affect the central nervous system (causing meningitis, in particular), and they remain life-threatening conditions [4,5]. BCG vaccination provides some protection against severe disseminated TB in childhood, but this protection is incomplete [42]. The development of antibiotics has greatly decreased childhood mortality due to TB, but more than 80 000 children still die from TB each year [43]. One of the fundamental unresolved questions in the field of childhood TB therefore concerns the nature of the predisposition to the development of severe clinical forms in only a minority of infected children. The findings obtained in the last decade have provided the first clue to the riddle, by showing that at least some cases of severe TB can be explained by single-gene inborn errors of immunity.

The first molecular evidence that childhood TB might reflect a Mendelian predisposition came from the observation of severe TB in children with classical primary immunodeficiencies (PIDs) [44]. In particular, a substantial number of children with chronic granulomatous disease (CGD) were diagnosed with severe TB in several countries [45–49]. In a recent survey investigating the occurrence of mycobacterial diseases in CGD patients, TB was found to be rather common in patients living in countries in which TB is endemic [50]. However, CGD is a rare disorder characterized by a high prevalence of multiple infectious diseases. Further progress towards an understanding of the genetics of severe TB came from the study of the syndrome of Mendelian susceptibility to mycobacterial diseases (MSMDs), which is defined by a selective vulnerability to weakly virulent mycobacterial species, such as BCG and environmental mycobacteria [51]. MSMD patients also often suffer from non-typhoidal, extra-intestinal salmonellosis. Since 1996, germline mutations in seven autosomal (*IFNGR1*, *IFNGR2*, *IL12B*, *IL12RB1*, *STAT1*, *IRF8, ISG15*) and two X-linked (*NEMO*, *CYBB*) genes have been discovered in MSMD patients [8,52–55]. High levels of locus and allelic heterogeneity have resulted in the definition of 17 different disorders, accounting for about half the known cases [56]. These defects are physiologically related, as they all result in an impairment of IFN-γ immunity. Several MSMD patients, particularly those with IFN-γR1 [57,58] and IL-12p40 [59] deficiencies, have been shown to suffer from infections due to both weakly virulent mycobacteria and *M. tuberculosis,* raising the question as to whether the TB observed in these patients could also be attributed to a monogenic predisposition.

The first answer to this question came when several siblings of MSMD patients carrying the same genetic defect as the index case were found to display severe TB as their sole infectious phenotype. This situation was initially observed in a child with partial IFN-γR1 deficiency, who was a sibling of an MSMD patient [60], and was subsequently observed in a male subject from a large multiplex X-linked kindred carrying a specific mutation of *CYBB* impairing the IFN-γ-dependent respiratory burst in macrophages [53]. However, the most common genetic defect identified in patients with severe TB to date is complete IL-12Rβ1 deficiency [61]. In one family, an IL-12Rβ1-deficient sister of a patient with MSMD developed abdominal TB [62]. Several children with severe TB and complete IL-12Rβ1 deficiency in the absence of a familial history of infections with weakly virulent mycobacteria have been identified [63,64]. In a more systematic search for *IL12RB1* mutations in 50 children with severe TB, two patients (4%) with complete IL-12Rβ1 deficiency were identified [24]. Overall, these results provided proof of principle for monogenic predisposition to severe TB, and raised the possibility that a substantial proportion of children with severe TB carry single-gene inborn errors of immunity. This proportion has been estimated at up to 45% by theoretical calculations [23], and can now be determined experimentally, by whole-exome and whole-genome sequencing. These findings have already paved the way for new treatments based on physiopathology. While until recently it was difficult to envision how point-of-care genotyping could be implemented in TB diagnosis, recent advances in hand-held PCR technology now suggest patient genotyping as a viable tool even in low- and middle-income countries [65,66]. The best example is provided by patients with IL-12Rβ1 deficiencies presenting TB owing to impaired IFN-γ production, for whom treatment with recombinant human IFN-γ, in addition to antimycobacterial drugs, has been shown to be effective [67].

## 4. Genetic control of pulmonary tuberculosis

Most people infected with *M. tuberculosis* present LTBI and do not develop primary TB (figure 1). Epidemiological studies have indicated that approximately 5–10% of individuals with LTBI go on to develop active TB during their lifetime, this risk decreasing with increasing time since infection [7,68]. Molecular epidemiology studies have shown that active TB due to reactivation of the original strain can occur decades after the initial infection [69]. The progression of infection within a subject from LTBI to pulmonary TB (PTB) reflects an impairment of host resistance to *M. tuberculosis*. This process may be triggered by acquired immunodeficiency, such as HIV infection or anti-TNF treatment. However, in subjects without overt immunodeficiency, the pathogenesis of reactivation remains unclear. As mentioned above, there is strong evidence that the development of PTB is influenced by host genetic factors. This genetic control is also likely to be different from that involved in primary TB [23,70]. Indeed, the study of genetic susceptibility to adulthood PTB has proved more difficult than that of susceptibility to severe childhood TB. In particular, no variants of genes from the IL12/IFN-γ circuit have been convincingly associated with PTB as yet. Current knowledge resulting from attempts to identify the genetic variants associated with PTB points to underlying heterogeneity, possibly due, at least in part, to the long-standing, multistep relationship between *M. tuberculosis* and its human host, through the natural history of PTB disease [71,72]. In particular, it is possible that specific subgroups of individuals with PTB have certain genetic risk factors, whereas other subgroups have other genetic risk factors. Such subgroups could potentially be defined on the basis of individual factors, such as clinical characteristics, or extrinsic factors, such as pathogen variability.

The vast majority of studies conducted to date to determine the molecular basis of PTB susceptibility have been association studies investigating the role of specific candidate genes. Most classical genetic association studies investigating PTB have focused on candidate genes, and a number of common risk variants have been reported in particular in immunity-related genes such as those encoding DC-SIGN, Toll-like receptors 1 and 2, vitamin D receptor, TNF, IL-1β or some HLA class II molecules [73]. However, there has been a lack of consistency between most of the reported results of independent studies [73,74]. One of the most convincing findings was the identification of associated polymorphisms of the *NRAMP1* gene (alias *SLC11A1*), the human orthologue of the murine *Nramp1* gene [17]. Following the initial association reported in a Gambian population [75], a meta-analysis of a large number of studies showed that several *NRAMP1* polymorphisms were significantly associated with PTB in African and Asian populations, but not in populations of European descent [76]. Two studies also provided evidence of a role for *NRAMP1* in early-onset TB. The first showed significant linkage to the *NRAMP1* gene in a large aboriginal Canadian family in which an outbreak of TB occurred [77]. In a follow-up study, common polymorphic alleles of *NRAMP1* were shown to be strong risk factors for TB (OR = 3.13 (1.54–6.25)) in children living in Texas, mostly of Hispanic and African origin, resulting in an allelic association acting in the opposite direction to that observed in adults [78]. These observations are consistent with the hypothesis that *NRAMP1* polymorphisms affect the speed of progression from infection to TB disease, accounting for the high frequency of some common alleles in patients with pediatric disease and the paucity of patients with the same alleles among TB cases with disease onset during adulthood. Overall, these studies provide strong support for a role of *NRAMP1* in TB, with an effect that is heterogeneous across populations, epidemiological settings and clinical phenotypes. They also highlight the importance of considering age at TB onset in these analyses. Generally, in the field of infectious diseases, stronger genetic effects are more pronounced in early-onset cases than in late-onset cases [70]. For example, in leprosy the association of genetic variants with disease has been shown repeatedly to be highly age-dependent [79,80].

The concept of stronger genetic effects associated with early-onset disease was further supported by the positional cloning of the first major locus conferring predisposition to PTB, which was found to be linked to chromosome region 8q [81]. Refined association mapping of the linked region identified variants of the *TOX* gene as strongly associated (OR = 3.09(1.99–4.78)) with the development of early-onset PTB (before 25 years of age) in populations from Morocco and Madagascar [82]. *TOX* encodes a nuclear factor involved in the development of T cells [83], particularly the CD4^+^ T cells [84,85] critical for immunity to mycobacteria [20]. Conversely, GWASs on PTB, considered as a single phenotype, have met with limited success to date. A first GWAS performed on a large sample from Gambia and Ghana identified a single SNP with a weak effect (OR = 1.19 (1.12–1.26)) located in a ‘gene desert’ on chromosome 18 as a risk factor for PTB [86]. Further imputation of the original Ghanaian data identified a second locus on chromosome 11p13 as protective against TB [87]. The main protective SNP allele was well replicated in the Gambian sample but displayed only borderline associations in Indonesian patients and in a very large sample (more than 10 000 subjects) of subjects from Russia [87]. Another GWAS recently conducted in a South African population confirmed the protective effect of the chromosome 11p13 factor but identified no new risk loci of GW significance [88]. Finally, a GWAS in Asian populations identified an independent TB risk locus in chromosome region 20q12, only in patients defined as ‘young cases’ (OR = 1.73 (1.42–2.11)) [89]. Based on the observed age distribution of this latter study, young cases were defined as having an onset of TB before the age of 45 years, although more refined analyses with lower age thresholds showed that higher deviations of *p*-values from the null hypothesis were observed with younger age cut-offs despite the lower number of cases in each subset [89]. Overall, a striking feature of these GWASs is the lack of replication of the PTB susceptibility factors previously detected in candidate gene analyses [8,73,74]. Overall, the GWAS results suggest that common variants may have a limited impact on predisposition to adult PTB, at least when considered as a single phenotype, and point to underlying heterogeneity, possibly in phenotype definition.

## 5. Perspectives

TB was long considered to be purely infectious, but there is increasing evidence to suggest that this disease also reflects host genetic vulnerability. However, the precise nature of the genetic factors involved remains largely unknown. Several non-mutually exclusive explanations can account for the difficulties experienced in identifying the causal variants, especially in GWASs. Genetic heterogeneity may play a role, together with a complex mode of inheritance involving incomplete penetrance and modifier genes [90,91]. Likewise, the contribution of rare variants to TB pathogenesis is attracting attention for two main reasons: (i) conceptually, rare variants bridge the gap between Mendelian and complex inheritance, and may account for the major loci identified through linkage studies [56,70,92]; (ii) experimentally, these variants abound in the genome and can now be studied by whole-exome and whole-genome sequencing [93,94]. However, the phenotypic heterogeneity of TB-related traits causes more serious problems. For example, three measurements of immune reactivity are currently used to detect infection with *M. tuberculosis*. However, the results of these tests display limited concordance, and each assay captures a different aspect of the antimycobacterial response [27]. MSMD probably provides the best example of the critical interplay between phenotype and genetic control. The syndromic phenotype is MSMD, but the identification of the underlying genetic defects was greatly facilitated by establishing endophenotypes (e.g. specific patterns of cytokine production such as IL-12 and IFN-γ [95]), guiding subsequent genetic analysis. Endophenotypes are more closely related to gene function and are likely to be generally useful for the dissection of TB phenotypes. In PTB, the most commonly used phenotype-defining characteristic is the presence of *M. tuberculosis* in the sputum of patients, regardless of the other clinical, microbiological and demographic covariables. This approach completely ignores the dynamic nature of pulmonary TB, the likelihood of different stages of this process being under different genetic controls, as shown in a mouse model of BCG infection [96], and the possible impact of the *M. tuberculosi*s strain on LTBI and clinical outcome [97–99]. The need for improvements in the definition of a more homogeneous and more refined TB phenotype for genetic studies is also demonstrated by the strong impact of age at onset of disease on our ability to detect genetic effects in clinical TB [78,82].

Traditional genetic approaches will also benefit from a better understanding of the molecular mechanisms of TB pathogenesis throughout the infectious cycle. For example, transcriptomic studies, including the search for loci involved in the expression of genes and/or microRNAs (expression quantitative trait loci, eQTL) in cell- or tissue-specific studies [100], based on RNA-seq in particular [101], are of major importance. A transcriptomic analysis of peripheral blood cells identified a neutrophil-driven IFN-α/β-inducible transcript signature in individuals with active PTB [102]. This blood signature was validated in independent studies [103–105], and was shown to be different from that of several infectious and pulmonary diseases [102,106], providing new insights into the most relevant pathways and candidate biomarkers for investigation in TB [107]. Some eQTLs associated with variation in gene expression levels in dendritic cells infected with *M. tuberculosis* [108] have also provided interesting candidate loci that remain to be tested for association with TB infection phenotypes. It is also possible that somatic mutations and epigenetic effects have a substantial impact on clinical susceptibility to TB, particularly in patients with late-onset disease [70]. Epigenetic mechanisms play a critical role in the immune system [109], and their investigation, particularly through GW analyses of the methylome [110,111], should provide new insight into the genetic basis of infection and clinical TB [112]. These epigenetic factors may be influenced by environmental and/or other host factors, such as diet, vitamin status or ageing [113,114], leading to additional possible gene × environment interaction effects. One of the main challenges in the next few years will be the integrated analysis of all these different sources of genomic information [115,116]. Finally, studies of various animal models, including zebrafish [90,91], rodents [15,16,117] and non-human primates [117,118], will be essential to provide critical information complementary to that obtained in human studies [7].

In the principal countries in which TB is endemic, disease control is based largely on passive case identification and drug treatment. This approach has proved effective for decreasing case mortality and human suffering, but the impact of current TB control on global TB trends is less clear [119–121]. The emergence and spread of extremely drug resistant strains resistant to the antibiotics currently available are warning signs that additional approaches will be required to halt endemic TB [122]. The availability of an effective vaccine against post-primary TB would provide us with a useful tool for decreasing TB transmission. Unfortunately, recent vaccine trials have yielded disappointing results [123,124], possibly reflecting our incomplete understanding of intrinsic vulnerability to TB as (i) mice that are genetically more resistant to *M. tuberculosis* infection benefit more from BCG vaccination than their susceptible counterparts [125], (ii) antimycobacterial immunity in humans is highly heritable and, thus, has a strong genetic component [30–32] and (iii) TB patients are more likely to suffer subsequent episodes of TB than would be expected on the basis of population incidence, strongly suggesting that TB patients have a susceptibility that cannot be overcome by conventional vaccination and/or antibiotics [126]. The strong positive selection of T-cell epitopes by *M. tuberculosis* across clinical strains presents an additional hurdle for vaccine development [127]. Taken together, these experimental data and observations *in natura* suggest that any effective TB vaccine must take into account the genetic susceptibility of patients if it is to trigger a protective response. Likewise, new therapeutic approaches based on the complementation of a specific immunodeficiency identified by human genetics should also have a positive complementary impact on the classical treatment of TB. For example, young patients developing disseminated TB during primary infection owing to the impaired production of IFN-γ (such as those with *IL12RB1* mutations) would benefit from targeted treatment with recombinant IFN-γ [67]. Finally, the recent discovery of *TST1* as a locus with an impact on intrinsic resistance to infection with *M. tuberculosis* provides another example for the usefulness of human genetics in TB control [39]. Once identified, the molecular mechanisms underlying *TST1* might constitute attractive targets for the prevention of infection by drug-based or vaccine interventions. The prevention of infection is the gold standard for stopping the TB endemic and studies of host genetics are an important weapon in the war against TB.
